# Leveraging immune checkpoint inhibitors in lung cancer patients with pre-existing autoimmune disease: clinical insights, optimal timing, and predictive biomarkers for optimal treatment outcomes

**DOI:** 10.3389/fimmu.2025.1539260

**Published:** 2025-05-21

**Authors:** Hui Yang, Sheng Yu, Hong Ge

**Affiliations:** ^1^ Department of Radiation Oncology, The Affiliated Cancer Hospital of Zhengzhou University & Henan Cancer Hospital, Zhengzhou, China; ^2^ Laboratory of Radiation Oncology, The Affiliated Cancer Hospital of Zhengzhou University, Zhengzhou, China

**Keywords:** lung cancer, immune checkpoint inhibitors (ICBs), immune-related adverse events (IRAE), autoimmune disease (AD), flare, predictive biomarker

## Abstract

The use of immune checkpoint inhibitors (ICIs) has revolutionized cancer treatment, particularly in lung cancer. However, their use in patients with pre-existing autoimmune diseases (PADs) presents unique challenges. PADs, such as rheumatoid arthritis (RA), systemic lupus erythematosus (SLE) and psoriasis, complicate the clinical management of lung cancer due to concerns about worsening autoimmune symptoms during ICI therapy. This review summarized the safety and efficacy of ICIs in lung cancer patients with PAD, focusing on the available clinical evidence, the optimal timing of ICI initiation, and the potential predictive biomarkers for immune-related adverse events (irAEs). Future prospective studies are needed to establish definitive guidelines for the use of ICIs in this population, with a focus on identifying patients at risk, managing ICI resumption after irAE and developing new medications with durable control of both cancer and PAD.

## 1 Introduction

Immune checkpoints (ICs) are pivotal in maintaining immune homeostasis and self-tolerance, balancing protective immunity against overactivation of the immune system. Recently, inhibitory ICs contribute to the immune escape of cancer cells and offer several therapeutic targets. Immune checkpoint inhibitors (ICIs), which target ICs, such as cytotoxic T-lymphocyte antigen 4 (CTLA-4), programmed death protein 1 (PD-1) or programmed death protein ligand 1 (PD-L1), effectively release the brakes on ICs and restore the anti-tumor immunity (1, 2).

Meanwhile, ICIs stimulate an excessive immune response, disrupt self-tolerance, and precipitate in immune-related adverse events (irAEs) that may affect nearly any organ system. The incidence of any grade irAEs ranges from 20% to 50% of patients, with most being mild to moderate in severity. Fatal irAEs are rare, occurring in 0.3-1.3% of patients, with myocarditis being the most common cause, followed by pneumonitis. Although the exact pathophysiological mechanisms remain unclear, irAEs are generally attributed to widespread immune activation, which can attack normal organs and manifest symptoms resembling autoimmune diseases (ADs) (3).

To date, the US Food and Drug Administration (FDA) has approved various PD-1/PD-L1 inhibitors and CTLA-4 inhibitors for cancer treatment, including in lung cancer patients. However, research specifically addressing cancer patients with pre-existing autoimmune diseases (PADs) remains limited. These patients are routinely excluded from clinical trials due to heightened concerns about the risk of irAEs and PAD flares.

Lung cancer is the most prevalent malignant tumor, with 14% to 25% of patients reported to have PADs at diagnosis. With the increasing use of ICIs and the significant proportion of lung cancer patients affected by PADs, it is important to assess the safety and efficacy of these treatments in this unique subgroup. While several studies have examined ICI safety and efficacy in general cancer populations (4–6), a comprehensive synthesis of recent evidence specifically focused on lung cancer remains notably absent from the literature. This gap is particularly significant given the unique immunobiological characteristics of pulmonary malignancies and their distinct response profiles to immunotherapy. While strong evidence from prospective studies is lacking, numerous case reports and retrospective studies offer limited insights into the use of ICIs in patients with PADs.

This review synthesizes current evidence on the use of ICIs in lung cancer patients with PAD, focusing on their safety and efficacy. It also explores the optimal timing for ICI administration and evaluates potential blood-predictive markers for irAEs. The review aims to deepen the understanding of ICI use in this unique population, thereby guiding treatment strategies and future research directions.

## 2 Lung cancer patients with PAD: epidemiology and mechanisms

**Figure 1:**
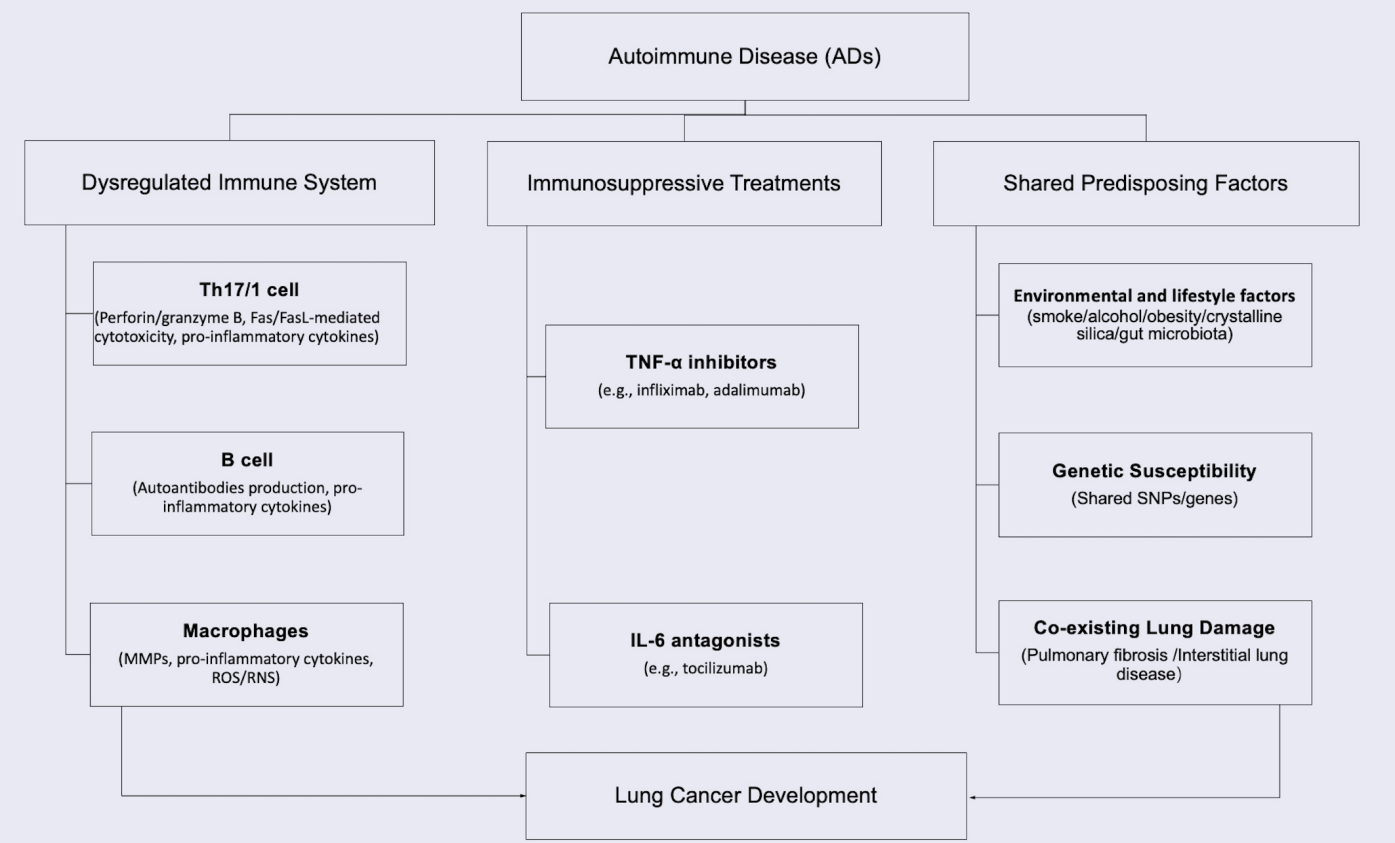
Pathogenic mechanisms linking PADs to lung cancer development.

The intersection of cancer and ADs represents a significant clinical challenge, with 11-25% of cancer patients having PADs (7). In lung cancer specifically, 14-25% of patients are diagnosed with PAD before cancer diagnosis (8). The bidirectional relationship stems from shared pathogenic mechanisms: dysregulated immunity promotes both autoimmunity and carcinogenesis, while immunosuppressive therapies impair tumor surveillance (9–11). Studies confirm organ-specific cancer risks associated with autoimmune and inflammatory diseases (9, 12–16), particularly for lung cancer in systemic sclerosis (SSc) (4.2-fold risk) (17), rheumatoid arthritis (RA) (1.7-fold) (13, 18), systemic lupus erythematosus (SLE) (1.6-fold) (19). Key mechanisms include immune system dysregulation, chronic tissue inflammation and damage caused by immunity overaction, immunosuppressive treatments, and the shared predisposing factors, as summarized in **Figure 1** (10–13, 20–25). Even when not associated with ADs, the tumor may coexist during either the active or stable phase of the disease (26).

### 2.1 Dysregulated of immune system, chronic inflammation and tissue damage

**Figure 2:**
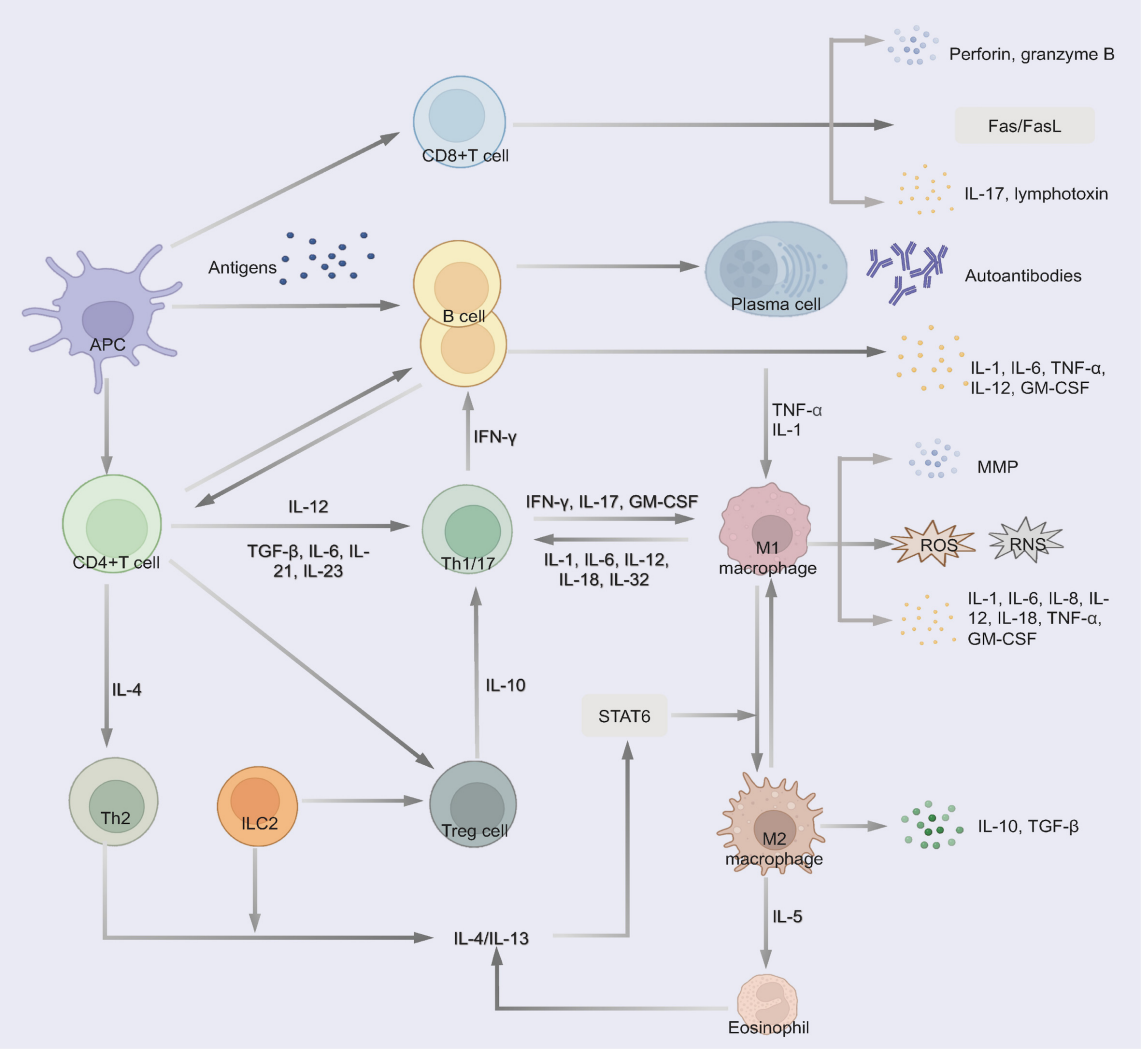
Immune dysregulation pathways in autoimmune diseases contributing to chronic inflammation and tissue damage. 1) CD4(+) T cells differentiate into Th1/17 cells and produce excessive pro-inflammatory cytokines. 2) Cytotoxic CD8+ T cells produce perforin and granzyme B, induce Fas/FasL-mediated cytotoxicity and the secretion of pro-inflammatory cytokines, thereby contributing to tissue damage. 3) B cells differentiate into plasma cells and secrete autoantibodies to trigger tissue damage through complement fixation and ADCC. Some B cells secrete multiple pro-inflammatory cytokines. 4) Macrophages polarize to a pro-inflammatory phenotype and release MMP, pro-inflammatory cytokines and small-molecule mediators of inflammation (such as ROS and RNS). Th2, ILC2 and eosinophil regulate the differentiation of macrophages to the M2 phenotype, which could secret anti-inflammatory cytokines. 5) Treg secret anti-inflammatory cytokines to suppress pathogenic T cells.

ADs create a chronically inflamed microenvironment that promotes lung carcinogenesis through multiple interconnected mechanisms (**Figure 2**). Dysregulated immunity disrupts self-tolerance with effector T cells (Th1/Th17), B cells, and macrophages sustaining a pro-tumorigenic cascade (27).

Th17 differentiation (driven by IL-6, TGF-β, and IL-23) and Th1 responses generate excess IL-17, IFN-γ, and TNF-α, activating macrophages and recruiting cytotoxic CD8(+) T cells and B cells (28–30). These CD8+ T cells exacerbate tissue damage via perforin/granzyme B and Fas/FasL-mediated cytotoxicity while secreting pro-inflammatory cytokines (30, 31).

B cells could contribute through autoantibodies production (via CD4+ PD1+CXCR5- cells peripheral Tph-cell) (32), triggering complement fixation and antibody-dependent cytotoxicity (ADCC) or pro-inflammatory cytokines secretion (IL-1, TNF-α) to promote inflammation, induce tissue damage, and activate oncogenic pathways (32, 33).

While macrophages release matrix metalloproteinase (MMP), pro-inflammatory cytokines (IL-1, IL-6, IL-8, etc.) and reactive oxygen species (ROS)/reactive nitrogen species (RNS) that induce DNA damage and genomic instability (34, 35). Otherwise, the induction of anti-inflammatory cytokines, such as TGF-β and IL-10, is reduced (35, 36).

M2 macrophages secrete IL-10 and TGF-β, while Th2/ILC2-derived IL-5 promotes M2 polarization, suppressing M1-mediated inflammation (33). This M1/M2 imbalance contributes to both AD pathogenesis and cancer development (37–39). Concurrently, ADs exhibit impaired Treg function, leading to defective immune regulation and uncontrolled tissue damage (39). Notably, chronic upregulation of immune checkpoints (PD-1/CTLA-4) in ADs may paradoxically facilitate immune evasion by pre-malignant cells (40, 41).

This vicious cycle of tissue damage, defective repair, and impaired surveillance creates an ideal niche for malignant transformation.

### 2.2 Immunosuppressive treatments

Immunosuppressive therapies play a crucial role in managing ADs but carry significant oncogenic risks. Widely used biologic agents including TNF-α inhibitors (e.g., infliximab, adalimumab) and IL-6 antagonists (e.g., tocilizumab) exert broad immunosuppressive effects that compromise critical antitumor immune mechanisms (42, 43). By inhibiting key inflammatory pathways, these treatments impair dendritic cell maturation, CD8+ T cell cytotoxicity, and NK cell surveillance - creating permissive conditions for malignant cell escape and proliferation. Clinical studies demonstrate that prolonged use (>2 years) of immunosuppressive therapies correlate with a 1.3-2.5-fold increased malignancy risk, particularly for non-melanoma skin cancers and lymphoproliferative disorders (42). This risk-benefit paradox necessitates careful monitoring of AD patients receiving chronic immunosuppressive therapy, particularly those with additional cancer risk factors such as smoking history or family predisposition (43).

### 2.3 Shared predisposing factors

Growing evidence highlights overlapping etiological factors between ADs and lung cancer. Genetically, shared signatures include susceptibility single nucleotide polymorphism (SNP) (rs13194781, rs1270942) linking SLE to lung carcinogenesis (44), as well as ferroptosis-related genes (FANCD2, HELLS, VLDLR) shared by RA and lung cancer (45). The immune-regulator PDE4A further demonstrates mechanistic convergence in lung cancer and multiple sclerosis (MS) (46).

Environmental and lifestyle factors synergistically promote both disease groups. Smoking, the predominant risk factor for lung cancer, concurrently activates pro-inflammatory pathways implicated in RA, SLE, SSc, and MS development (22, 47). Additional shared risks include alcohol consumption, obesity, occupational exposures (e.g., crystalline silica) (16, 18). The gut-lung axis further connects these conditions, where microbiota dysbiosis impairs immune homeostasis and facilitates IL-25-mediated recruitment of inflammatory lymphoid cells to pulmonary tissues (48–50).

Pulmonary complications of ADs substantially elevate cancer risk. Chronic inflammation in SSs and RA drives pulmonary fibrosis, a condition characterized by thickened and scarred lung tissue (13, 14), inducing hypoxia and mutagenesis that predispose to malignant transformation (23). Notably, patients with scleroderma-associated interstitial lung disease (ILD) exhibit exceptional vulnerability to lung cancer, underscoring the need for vigilant monitoring in this population (24, 25).

## 3 Clinical evidences for ICIs in lung cancer patients with PAD: safety and efficacy

ICIs are becoming a common and effective way to fight lung cancer through immune regulation. Up to 80% of patients treated with ICIs develop irAEs, with the first irAEs occurring 3–6 months after the start of ICI treatment (51–53). IrAEs can affect any organ but most commonly affect the skin, gastrointestinal tract, liver, lungs and endocrine glands. Although irAEs are usually mild and can be managed by clinicians, few can be life-threatening (54). The most common serious irAEs are pneumonitis and colitis, while myocarditis is the irAE with the highest mortality rate (53, 55).

Although the pathogenesis of irAEs is still not fully understood, the over-activation of immunity is very similar to ADs. IrAEs may develop through a combination of pathways involving autoreactive T cells, autoantibodies, and cytokines. Briefly, ICI therapy inhibits the combination of ICs and their gland, thus removing the inhibitory signals and causing aberrant activation of T cells, further leading to the production of autoantibodies and inflammatory cytokines, regulating immune cells and finally contributing to the development of irAEs (55, 56).

Cancer patients with PAD were thought to be at increased risk of flares or *de novo* irAEs following ICI treatment due to their pre-activated immune system (57) and were excluded from clinical trials. Given the increasing global incidence and prevalence of ADs worldwide, this raises important questions about the safety and efficacy of ICI treatment in these patients (58). However, their absence from clinical trials makes it difficult to consider the safety and efficacy of ICIs in this population (59).

The association of PAD with the risk of irAEs and survival has been controversial in various studies. Previous studies have shown that in patients with PAD treated with ICIs, 25-50% develop PAD flares, while approximately 16-40% develop *de novo* irAEs, and 24-46% discontinue ICIs due to irAEs (60, 61). Analyses of retrospective studies and the SEER database suggest that the development of flare or *de novo* irAEs after ICI initiation is more common in patients with PAD (62–64). A meta-analysis combining 95 studies with 23,897 patients has reported that patients with PAD were 30% more likely to report an irAE than patients without PAD (61). However, some studies have shown that there is no association between PAD and the incidence or severity of irAEs (65, 66). In terms of efficacy, some studies have even reported that patients with PAD had an increased progression-free survival (PFS) or overall survival (OS) when treated with ICIs compared to patients without PAD (63–65). In the meta-analysis, there were no statistically significant differences in cancer response to ICIs between patients with and without PAD (61).

A meta-analysis focusing on lung cancer included 250 non-small cell lung cancer (NSCLC) patients with PAD from 9 cohort studies (67). The incidence of PAD flare was 23% (95% CI: 9%-40%). PAD was associated with an increased risk of any grade and grade 3–4 *de novo* irAE. More discontinuations were observed in patients with PAD (10% vs. 3%). PAD was also associated with a better tumor response (complete response/partial response). However, this meta-analysis included three studies with interstitial lung disease (ILD) patients, those who may not be AD patients. Due to the small sample size and a high heterogeneity, the safety and efficacy of ICIs in lung cancer with PAD may be different from the results in all cancer populations. Without sufficient clinical evidence, it is difficult for clinicians to make decisions about how to treat this unique patient population.

To illustrate the use of ICI in lung cancer patients with PAD, we conducted a comprehensive search of MEDLINE, EMBASE, Web of Science, PubMed, and the Cochrane Library databases for studies of NSCLC patients with PAD who received ICI treatment. After removing studies with no usable data and duplicate studies, we identified 19 retrospective studies (68–87). We did not include the case reports or case series to avoid bias. Eleven studies are specific to lung cancer (68, 69, 72, 73, 75, 77, 79, 81–84, 87), and seven studies have more than one type of cancer from which we can get data on lung cancer (70, 71, 74, 76, 78, 80, 85, 86). Of these, 13 studies with 468 patients were able to provide safety data and 15 studies with 997 patients provided efficacy data. We have summarized the safety and efficacy data in **Table 1**.

**Table 1:** The safety and efficacy data of ICIs in lung cancer patients with PAD.

Study	ICI target	Duration	Cancer	No.	Flare		*De novo* irAEs		ICI discontinuation	ORR	DCR	mPFS (months)	mOS (months)
					Any grade	≥grade 3	Any grade	≥grade 3					
Leonardi 2018 (68)	PD(L)‐1	2015-2017	NSCLC	56	23.2%	3.6%	37.5%	10.7%	14.3%	22%	53.1%	–	–
Pease 2018 (69)	PD-1	2015-2016	NSCLC	9	11.1%	–	–	–	–	–	–	–	–
Cytryn 2019 (70)	NA	2011-2018	Lung cancer	29	27.6%	NA	34.5%	6.9%	10.30%	–	96.0%	6.0	8.5
Khozin 2019 (72)	NA	2011-2018	NSCLC	538	–	–	–	–	–	–	–	4.2	12.4
Tison 2018 (71), 2019 (73)	PD(L)‐1	2017-2018	NSCLC	40	–	–	–	–	–	53.8%	–	11.8	22.4
Alexander 2021 (74)	PD(L)‐1	2015-2018	Lung cancer	11	–	–	–	–	–	–	–	5.3	12
Calvo 2021 (76)	PD-1	2016-2018	NSCLC	6	33.3%	–	–	–	–	–	–	–	–
Debieuvre 2021 (77)	PD-1	2016-2019	NSCLC	60	–	–	35.0%	8.3%	10.00%	–	–	–	11.3
Fountzilas 2021 (78)	PD(L)‐1 , CTLA-4 and combination	2014-2021	NSCLC	77	–	–	–	–	–	–	–	16.5	29.1
Sawhney 2021 (79)	NA	2017-2020	NSCLC	8	12.5%	0	12.5%	12.5%	NA	71.4%	85.7%	–	–
Zhang 2021 (80)	PD(L)‐1	2015-2018	Lung cancer	17	5.9%	–	23.5%	–	0	23.5%	–	–	–
Ardizzoni 2021 (75) and 2022 (81)	PD-L1	2017-2018	NSCLC	30	–	–	13.30%	3.3%	6.70%	–	–	2.9	10.1
Higgins 2022 (82)	PD-1	2014-2019	Lung cancer	19	–	–	–	–	–	–	–	5.5	17.2
Ohe 2022^a^ (83)	PD-L1	2018	NSCLC	27	–	–	33.80%	7.4%	–	–	–	–	–
Ohe 2022^b^ (83)	PD-L1	2018	NSCLC	98	–	–	32.30%	8.2%	–	–	–	–	–
Ansel 2023 (84)	PD-1	2017-2018	NSCLC	10	30.0%	10.0%	90.0%	10.0%	10.0%	–	–	–	–
Reid 2023 (85)	PD-1	2016-2019	NSCLC	25	23.0%	–	27.0%	4.0%	–	–	–	–	26.8
Reid 2023 (85)	PD-1+ CTLA-4	2016-2019	NSCLC	16	18.0%	–	71.0%	26.0%	–	–	–	–	–
Androdias 2024 (86)	PD(L)‐1	2019-2022	NSCLC	8	12.5%	–	–	–	–	0	50.0%	–	–
Asao 2024 (87)	PD-1	2010-2020	NSCLC	69	25.40%	–	45.10%	23.9%	38.0%	33.80%	59.1%	5.8	16.5

Ohe 2022^a^ (83) included 27 patients had a medical history of autoimmune disorders, and Ohe 2022^b^ (83) included 98 patients were experiencing an autoimmune disorder at ICI initiation.

The majority of patients had NSCLC, with only two patients having small cell lung cancer. The ICIs used in these studies include PD-1 inhibitors (nivolumab, pembrolizumab), PD-L1 inhibitors (atezolizumab, durvalumab), CTLA-4 inhibitor (ipilimumab), and the combination. In the 190 patients with detailed PAD data from seven studies (68, 70, 76, 79, 84, 86, 87), we find 50.0% of patients have rheumatological PAD, followed by endocrine PAD (18.4%), dermatological PAD (14.7%) and neurological (7.9%). RA is the most common PAD, occurring in 25.8% of patients (49/190), followed by psoriasis and thyroiditis. The grade ≥3 irAEs are colitis (n=1), diarrhea (n=1), hepatitis dysfunction (n=2), and pneumonitis (n=1).

### 3.1 Safety of ICIs in lung cancer patients with PAD

The rate of PAD flares was reported in 11 studies, ranging from 5.9% to 33.3%, and the incidence of grade ≥ 3 flares was 0-10.0%. The incidence of *de novo* irAEs of any grade in the 12 included studies ranged from 12.5% to 90.0%, with only two studies reporting an incidence greater than 50%. The incidence of grade ≥ 3 *de novo* irAEs was reported in 3.3%-26.0% of patients in seven studies and was less than 10.0% in 50.0% of studies. Permanent discontinuation of ICIs due to flares or *de novo* irAEs was reported in 6.7-38.0% of patients from six studies.

Some studies have reported that the risk and severity of irAEs in patients with PAD are similar to those in the overall population (78, 81). Higgins et al. reported that the PAD group tended to have a higher incidence of irAEs (42.9% vs. 32.7%), although not statistically significant (p = 0.130) (82). The incidence of grade ≥ 3 irAEs was reported to be unaffected by PAD (79, 87). Two studies even reported a lower incidence of grade ≥ 3 irAEs in the PAD group, although the difference was not significant (82, 83).

Any grade and grade ≥ 3 flare/*de novo* irAEs were not increased compared to patients without PAD. Ansel et al. showed that low-grade adrenal insufficiency was the only irAE that occurred significantly in the PAD group (p = 0.02) (84). Ardizzoni et al. found a moderate increase of respiratory or gastrointestinal AEs in patients with PAD (81). Ohe et al. reported that the incidence of grade ≥ 3 irAEs was higher in patients with a history of ILD (18.4% vs. 9.4%, p = 0.0056) or current ILD (24.6% vs. 8.9%, p < 0.0001) compared to the patients without ILD (83).

So far, based on the limited data, ICI treatment appears to be relatively safe for lung cancer patients with PAD. Larger trials could provide more details about which patients need to be monitored closely and what AEs may occur.

### 3.2 Efficacy of ICIs in lung cancer patients with PAD

Two multicenter studies reported that ICIs were beneficial in lung cancer patients with PAD compared with non-ICI treatments (87, 88). Among the included studies, the overall response rate (ORR) ranged from 22.0% to 71.4% in six studies, and the disease control rate (DCR) ranged from 50.0% to 96.0% in five studies. Median PFS ranged from 2.9 to 16.5 months in eight studies, and median OS ranged from 8.5 to 29.1 months in ten studies.

Ardizzoni et al. reported a slightly shorter median OS in the PAD group compared with patients without PAD (10.1 vs. 11.2 months) (81). Most studies reported that the presence of PAD did not affect survival (68, 72, 79, 88). Higgins et al. even showed that patients with PAD tended to have longer median PFS (5.5 vs. 3.5 months, p = 0.8551) and median OS (17.2 vs. 14.4 months, p = 0.991) (82). Data are still limited to answer the question of the efficacy of ICIs in lung cancer patients with PAD.

### 3.3 Combination therapy: ICI and chemotherapy

Asao et al. included 69 NSCLC patients with PAD and compared the safety and efficacy of ICI monotherapy and the combination with cytotoxic chemotherapy (87). PAD flares were more frequent with combination treatment than with ICI monotherapy, although the difference was not significant (35.7% vs. 22.8%, p = 0.32). There were no significant differences in the incidence of *de novo* irAEs between combination treatment and ICI monotherapy (combination vs. monotherapy: all-grade irAEs: 50.0% vs. 43.9%, p = 0.68; grade ≥ 3 irAEs: 21.4% vs. 24.6%, p > 0.99). PAD exacerbations were more likely when NSCLC was diagnosed within one year after the diagnosis of AD (p = 0.016). When adjusted by inverse probability weighting, the use of ICI could prolong survival in NSCLC patients with PAD (p = 0.0006).

Although the rates of flares and irAEs tended to be higher with combination therapy, the differences were not significant, supporting the feasibility of combining ICI and chemotherapy in patients with NSCLC and PAD. Physicians need to be cautious when treating patients within a short time after diagnosis of PAD.

### 3.4 Dual therapy: combination of PD-1 and CTLA-4 inhibitors

Reid et al. enrolled 25 lung patients treated with PD-1 inhibitors and 16 lung patients received the combination of PD-1 and CTLA-4 inhibitors (85). The dual therapy showed an increased incidence of PAD flares, and any grade/high grade *de novo* irAEs compared to PD-1 inhibitor alone, but was not significantly different (dual therapy vs. monotherapy: flares: 18% vs. 23%, p = 0.36; any grade *de novo* irAEs: 71% vs. 27%, p = 0.12; high grade *de novo* irAEs: 26% vs. 4%, p = 0.17).

Thus, the combination use of PD-1 and CTLA-4 inhibitors does not increase the risk of flare or *de novo* irAEs and could be used in lung cancer patients with PAD.

### 3.5 The association between irAEs and clinical outcomes

Recently, it has been suggested that irAEs may be associated with therapeutic response and may serve as survival predictor. Tison et al. found that flare/*de novo* irAE was associated with prolonged PFS in cancer patients with PAD treated with ICIs (p = 0.026) (73). In addition, Fukihara et al. reported that pneumonitis is associated with poor outcomes in NSCLC patients treated with PD-1 inhibitors (89). However, Cytryn et al. reported no statistically significant association between survival and the presence of irAE (70). The relationship between irAEs and clinical response has not been fully investigated. Further studies are needed to evaluate the correlation between the flares, irAEs, their severity, organ site and efficacy (90).

These studies have shown that ICIs are effective, often leading to durable responses with tolerable toxicity. Physicians should consider using ICIs in lung cancer patients with PAD. Combining an ICI with chemotherapy or using two types of ICI is also acceptable. Notably, the ongoing NCT03816345 phase Ib trial (National Cancer Institute (NCI)-sponsored) aims to enroll 300 patients with advanced cancer and PADs (RA, SLE, inflammatory bowel disease (IBD), SSc, psoriasis, etc.) to evaluate nivolumab safety and biomarkers (completion: 2026-08-31). This may provide the first high-quality data on irAE patterns in specific PADs (91). The use of ICIs in this population remains challenging and further prospective, controlled studies are needed.

## 4 Optimal timing of ICI treatment in lung cancer patients with PAD

To determine the optimal timing of ICI treatment, we need to balance clinical benefit and toxicity. Because of the underlying mortality associated with cancer, the short-term risk of death from cancer may outweigh the risk of worsening of AD or induction of irAEs. Patient assessment by a multidisciplinary team is required to make clinical decisions.

### 4.1 PAD status

We focused on the relationship between PAD status and the safety of ICI use. Patients can be divided into active status and inactive status. Leonardi et al. reported on 56 NSCLC patients with PAD who were treated with PD-1 inhibitors, and the patients with active PAD were the majority of those who experienced flares (7). Cortellini et al. showed that inactive patients had fewer irAEs and a better prognosis in cancer patients treated with PD-1 inhibitors compared to patients with active PAD (66). Two meta-analyses reported no differences in the presentation of flares/irAEs and ORR between patients with active and inactive PAD (92, 93). Khozin et al. also found there was no association between PAD status and outcomes in NSCLC patients but reported higher rates of selected AEs, including endocrine, gastrointestinal and hematological disorders, in patients with active PAD (72).

Therefore, it is generally safe to receive ICIs when PAD is well controlled. For those whose disease is not yet under control, there is a high likelihood of flares/irAEs. Disease status must be considered and carefully evaluated before starting treatment and during ICI therapy.

### 4.2 Baseline use of immunomodulatory medications

The management of immunomodulatory medications prior to ICI initiation requires careful consideration of both AD control and potential impacts on cancer outcomes. These agents can be categorized into three clinically relevant classes: (1) corticosteroids, (2) conventional disease-modifying antirheumatic drugs (DMARDs; e.g., methotrexate), and (3) biologics/targeted therapies (e.g., TNF inhibitors, IL-6 antagonists) (94). Current evidence suggests differential effects based on medication class.

Cytryn et al. (70) demonstrated that overall immunomodulator use at ICI initiation was not associated with increased AD flares/irAEs or survival differences, while Leonardi et al. (7) specifically found no association between immunomodulators (including both corticosteroids and steroid-sparing agents) and ICI response rates (p = 0.66). The meta-analysis found that these agents may paradoxically reduce severe irAE risk (29.4% vs 70.0%, p = 0.007) while maintaining comparable overall immunotoxicity profiles (43.2% vs 58.3%, p = 0.148) (95).

However, corticosteroid-specific analyses reveal significant concerns; Fountzilas et al. (78) reported significantly poorer PFS in patients receiving baseline corticosteroids (p = 0.003), a finding supported by two additional studies (73, 85) and confirmed in a recent pooled analysis of six clinical trials by Verheijden et al. (96), which showed reduced objective response rates (ORR: 28% vs. 42%) and shorter PFS (HR: 1.54) with corticosteroid use for treatment-related adverse events (trAEs).

Data on DMARDs and biologics remain limited but suggest intermediate effects. While they appear less detrimental than corticosteroids for ICI efficacy, their immunomodulatory mechanisms may still partially interfere with checkpoint blockade. Notably, the reduced severe irAE risk seen with immunosuppressive therapy overall may be particularly relevant for this class (95).

These findings indicate that while baseline immunomodulators—especially corticosteroids—may reduce ICI efficacy, they do not elevate flare risk and could potentially decrease severe immune-related toxicity. Consequently, clinical practice should prioritize: (1) initiating ICIs during AD quiescence, (2) minimizing pre-ICI corticosteroids to <10 mg/day prednisone-equivalent (preferably ≤7.5 mg/day for maintenance therapy (97), and (3) favoring steroid-sparing immunomodulators when continuous treatment is required. This strategy achieves optimal balance between preserving antitumor immunity and maintaining AD control.

### 4.3 PAD Type

Different PADs may pose different risks to patients receiving ICI treatment. Previous studies have found that flares are more common in patients with pre-existing psoriasis/psoriatic arthritis (39%), IBD (37%) and RA (36%) (63). Gutzmer et al. noted that PAD flares appeared to be more common in patients with rheumatological PAD and psoriasis (98). Alexander et al. found that patients with rheumatological PAD had a significantly higher incidence of flares than patients with non-rheumatological PAD (74). Patients with RA also have a higher incidence of all-grade and severe irAEs (65).

In our review, 50.0% of patients have rheumatological PAD, followed by endocrine PAD (18.4%), dermatological PAD (14.7%) and neurological (7.9%). RA is the most prevalent PAD, occurring in 25.8% of cases (49/190), followed by psoriasis and thyroiditis.

RA patients could be well-controlled by DMARDs, biologics, or low-dose steroids (99, 100). So, ICIs could be started in these stable patients stopped their DMARDs or controlled by low-dose. If RA flares occur post-ICI, they typically present as joint pain, swelling, or stiffness but are rarely severe or life-threatening. Flares can often be managed with corticosteroids, nonsteroidal anti-inflammatory drugs (NSAIDs), or escalation of RA therapy without requiring ICI discontinuation and did not appear to negatively impact the tumor response to immunotherapy (101, 102).

Psoriasis is often mild-to-moderate and well-controlled with topical agents, phototherapy, or systemic therapies (e.g., methotrexate, biologics) (103). ICI-induced psoriasis flares usually manifest as worsening skin plaques or new-onset psoriatic lesions, but severe cases (e.g., erythroderma, pustular psoriasis) are rare. Most flares respond to topical steroids, methotrexate, or biologics without needing ICI cessation (6, 103, 104).

Patients with thyroiditis always have stable thyroid function on hormone replacement (levothyroxine) or antithyroid medications. ICIs commonly induce hypothyroidism (more frequent) or transient hyperthyroidism, but thyroid storms or life-threatening dysfunction are rare (93). Monitoring TSH/T4 and adjusting thyroid medications usually suffice (105).

Since ICIs may be the most effective treatment option for advanced lung cancer, and even the only choice for those intolerance to chemotherapy and unsuitable to target treatment, the benefits often outweigh the risks of RA exacerbation, particularly when PADs are manageable with low-dose of medications. It is importance to individually assess the risk-benefit rather than blanket avoidance. Thus, physicians should consider the type of PAD when deciding whether to treat with ICIs, especially rheumatological PAD. Cancer patients with rheumatological PAD may require careful stabilization and possibly delayed use of ICIs.

### 4.4 ICI type

PAD flares were increased in patients receiving PD-1/PD-L1 inhibitors, while *de novo* irAEs were observed more frequently in patients receiving CTLA-4 inhibitors (95). Postow et al. reported that colitis and hypophysitis seem to be more common with CTLA-4 inhibitors, whereas pneumonitis and thyroiditis are more common with PD-1 inhibitors (51).

The risk of irAEs was lower in patients on monotherapy compared to combination therapy (85, 95). Reid et al. reported that patients with gastrointestinal PAD were more likely to experience flares with combination ICI therapy, and patients with dermatological or endocrine PAD had a lower incidence of *de novo* irAEs with monotherapy (85).

Therefore, physicians should be cautious when using a combination of PD-1/PD-L1 inhibitors and CTLA-1 inhibitors in patients with gastrointestinal, dermatological or endocrine PAD.

### 4.5 Cancer stage and progression

Patients with advanced or rapidly progressing lung cancer may need earlier ICIs to effectively control disease progression. In early, slow-progressing cases with PAD, delaying ICIs until the PAD is better controlled may be a safer approach (106).

In conclusion, the optimal timing of ICIs in lung cancer patients with PAD should be personalized based on the status and type of PAD, the use of immunoregulatory treatment, the type of ICI used, and the stage of lung cancer. It is generally recommended to start ICI treatment when PAD is stable. Minimal immunosuppression at the initiation of ICI must be achieved by stopping or minimizing the dose of medications in quiescent patients. It is important to use low-dose immunosuppression treatment to prevent flares and to avoid further delay of ICI initiation in some patients. The timing of ICI can be complicated by the high heterogeneity of PAD. Experienced multidisciplinary team discussions, involving oncologists, rheumatologists, and other specialists, are essential for optimal timing and management.

## 5 Predictive biomarkers of irAEs after ICI initiation

PAD flares or irAEs generally improve with the discontinuation of ICIs with or without the administration of immunosuppressive therapy. However, several case reports raise concerns about the potentially irreversible morbidity of irAEs. Prediction and early recognition are crucial to optimizing therapy, avoiding discontinuation of ICI, and preventing morbidity and mortality. Currently, there is no reliable biomarkers correlating with risk of irAEs. Considering blood as a non-invasive and convenient origin for tests. We focused on potential blood biomarkers based on cancer patients without PADs and summarized the advances.

### 5.1 Autoantibodies

Autoantibodies detected at the baseline and during treatment in cancer patients treated with ICI may predict the incidence of irAE (107). Sakakida et al. reported that positive anti-nuclear antibody (ANA) was associated with a higher risk of ICI-mediated colitis, but not with classic ANA-associated ADs such as SLE and scleroderma (108). Osorio et al. found that the baseline levels of anti-thyroglobulin and anti-thyroid peroxidase antibodies were higher in NSCLC patients who developed thyroiditis after the PD-1 inhibitor treatment (109). In a retrospective study of NSCLC patients treated with PD-1 inhibitors, pre-existing ANA, rheumatoid factor, anti-thyroglobulin and anti-thyroid peroxidase antibody positivity correlated with the development of irAEs, but also with clinical benefit from ICIs (110).

Another study reported that patients who experienced irAEs had a low baseline autoantibody level, which increased significantly after initiation of ICI therapy (111). This may suggest that the dynamic change may be more useful for the early recognition of irAEs.

The likely mechanism is that PD-1/PD-L1 blockade or deletion of PD-1 can led to increased B cell proliferation and antibody response to T cell-independent antigens (112), resulting in autoantibody expansion and subsequent irAE development. Monitoring the dynamic change of relevant autoantibodies at baseline will help to identify high-risk patients and detect the early onset of irAEs during ICI treatment.

### 5.2 Cytokines

As irAE is a result of over-activation of the immune system, baseline and dynamic changes in cytokines have been extensively studied to predict irAE. Lower baseline levels of TNF-α, IL-6, IL-8, IL-15, CXCL9, CXCL10, CXCL11, and CXCL19 and higher baseline levels of IL-17 or IL-6 are associated with a high risk of irAE (113–117). High levels of IL-17 (114) and IL-6 (115) were also found to correlate with high-grade irAEs. Baseline Ang-1 (p = 0.005) and CD40L (p = 0.006) were significantly increased in patients who developed dermatitis, compared to those who did not. Patients who developed pneumonitis had significantly elevated baseline IL-17 (p = 0.009) and trends towards decreased baseline IL-8 (p = 0.06) and IL-15 (p = 0.06). In patients who developed colitis, there was a trend towards decreased baseline GCSF (p = 0.08) (116).

Significant increases in the levels of IFN-γ, IL-6, GM-CSF, CCL5, CXCL9, and CXCL10 levels after treatment are associated with irAE (113, 115, 118, 119). Elevated levels of IL-6 are associated with severe irAEs, particularly colitis (115). IL-17 has been observed to be elevated in patients with ipilimumab-induced colitis (120). Significant downregulation of MICA, CX3-CL1 or VEGF-A is associated with dermatitis (116). Patients who developed pneumonitis had significant upregulation of MICB, IL-2, IL-17, IFN-γ and CCL4 (116).

### 5.3 Gene expression

Whole blood could provide gene expression information that associated with the risk of irAE. The SNP rs2910164 at baseline and during ICI therapy, which leads to reduced miR-146a expression, was associated with an increased risk of developing severe irAEs, and reduced PFS (121). Other SNPs, such as rs11743438 and rs3026321, mapped to genes that associated with inflammation and ADs and led to the development of irAEs (122). Friedlander et al. used whole-blood RNA transcript-based gene signatures and reported a 16-gene signature panel to detect severe colitis/diarrhea in patients with advanced melanoma treated with the CTLA-4 inhibitors (123). When using the next-generation sequencing technique to test the circulating tumor DNA in blood samples, the baseline expression of several immune-related genes, including CD3E, CD37, CD4, and IL-32, is associated with increased gastrointestinal irAEs. In particular, increased expression of CD177 and CEACAM1gene, two neutrophil-activation markers, were closely associated with gastrointestinal irAEs and early predictors (124). Researchers found gastric cancer patients with alterations in CEBPA, FGFR4, MET, or KMT2B gene had a greater likelihood of irAEs (p = 0.09) (125).

Since T and B lymphocytes are also important mediators of immune tolerance and play a crucial role in the occurrence of irAEs. Peripheral CD8+ T cell clonal expansion has been found to correlate with the development of severe irAEs in cancer patients treated with CTLA-4 inhibitors (126, 127).

### 5.4 Routine blood count

Routine blood count could provide information about immune conditions and serve as valuable indicators of irAE risk. High absolute lymphocyte count, low absolute neutrophil count, low absolute monocyte count, and low neutrophil-to-lymphocyte ratio (NLR) before and during ICI treatment are associated with irAE onset in cancer patients with ICI treatment (128). Pavan A et al. reported that low NLR and platelet-to-lymphocyte ratio (PLR) at baseline were significantly associated with the development of irAEs (129). A high absolute lymphocyte count has been associated with an increased risk of irAEs, possibly due to increased immune reactivity (130). A high level of baseline absolute eosinophil count is an indicator of ICI-associated pneumonitis (130). Increased total white blood cell count and decreased relative lymphocyte count are associated with severe irAEs or lung/gastrointestinal irAEs (131) and a significant increase of C-reactive protein is an early marker of irAE (132).

In summary, some results of the current studies are controversial, further research is needed to validate these biomarkers in large prospective trials and to develop predictive panels to stratify patients by risk in cancer patients with PAD undergoing ICI therapy.

## 6 Safe use of ICIs in lung cancer patients with PAD

### 6.1 Improving the treatment strategies

Oncologists should be familiar with the manifestations of ADs, risk factors for irAEs, and related therapeutic medications. A multidisciplinary team including rheumatologists, gastroenterologists, endocrinologists, neurologists and dermatologists is essential (56). Before ICI treatment, physicians must perform a baseline medical history, physical examination, appropriate laboratory tests, and imaging tests to assess the risk of flares/irAEs and determine the optimal timing of ICIs based on the cancer stage, PAD status (active/inactive) and type (mild/life-threatening), and the use of immunoregulatory treatments. Predictive biomarkers can be used to determine the likelihood of irAEs and the possible irAEs, and may also aid in early detection and intervention, which could reduce the duration and severity of irAEs (133).

**Figure 3:**
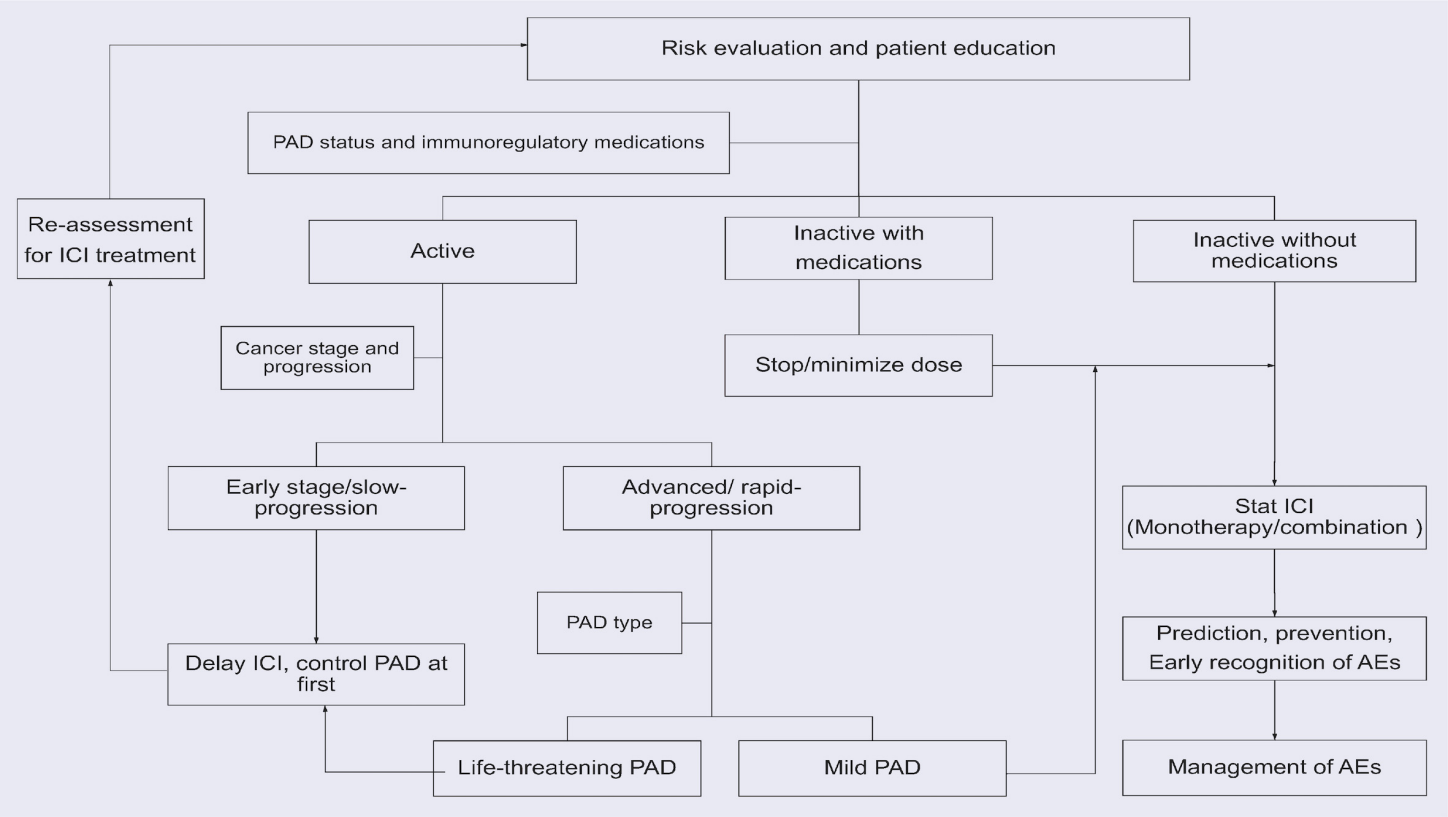
The recommended treatment process for lung cancer patients with PADs. The strategy may vary depending on the type of PADs.

Physicians need to discuss the symptoms and signs of possible irAEs with patients and their families. Education and communication will enable patients to recognize irAEs early and seek timely medical help, especially when patients are discharged from hospital. As there are no clear recommendations available, we recommend a treatment process for lung cancer patients with PAD is illustrated in **Figure 3**, the strategy may vary depending on the type of pre-existing autoimmune disease.

In inactive patients, ICI can be started after discontinuing or minimizing the use of immunoregulatory therapy. The type of ICI or its combination with chemotherapy or a different type of ICI should be discussed personally.

In patients with active PAD, clinicians should weigh the potential benefits of ICIs against the possible AEs. In advanced cancer patients with rapid progression, there may be no other options other than ICIs. If the PAD is mild with acceptable possible AEs, ICI should be used with close monitoring. If the PAD is also severe with life-threatening symptoms, proper control is required before immunotherapy can be started. For active patients with early or slowly progressing cancer, it’s better to delay the use of ICIs and control the PAD at first. Then, re-evaluate the possibility of using ICIs and use ICIs if the PAD becomes inactive.

Once ICI treatment is started, it is important to conduct regular examinations. Abnormal findings on relevant tests will help in the early detection of flares/irAEs. The management of irAEs in patients treated with ICIs should always be considered. There is no specific guideline for ICI treatment in cancer patients with PAD; we refer to the recommendations of the National Comprehensive Cancer Network (NCCN) (97), the Society for Immunotherapy of Cancer (SITC) (134, 135), the American Society of Clinical Oncology (ASCO) (136, 137), and the European Society for Medical Oncology (ESMO) (138) for the irAE management recommendations. IrAEs are graded from 1 to 5 according to the severity based on the Common Terminology Criteria for Adverse Events (CTCAE). Briefly, discontinuation of ICIs is not necessary for grade 1 irAEs if close monitoring is feasible, whereas temporary withholding of ICIs and moderate systemic corticosteroids should be considered for most grade 2 toxicities. ICIs are resumed when toxicity decreases to grade 1 or symptoms resolve. For grade 3 irAEs, ICIs should be discontinued and high-dose corticosteroids should be started and tapered over 4–6 weeks. If symptoms worsen, steroid-sparing biologic immunosuppressants such as TNF-α inhibitors or IL-6 antagonists should be considered. For grade 4 irAEs, permanent discontinuation of ICIs is recommended. With proper management, most irAEs in cancer patients with PAD resolve, and only few patients discontinue using ICIs.

### 6.2 Managing the resumption of ICIs after flares/irAEs

When the flares/irAEs improve with treatment, the physicians will consider resuming ICI treatment. Here we summarize the previous research and try to make some suggestions.

Allouchery et al. reported 180 patients from the French pharmacovigilance database who experienced at least one grade ≥2 irAE leading to ICI discontinuation; 41.4% were lung cancer patients (139). 61.1% of patients had no grade ≥2 irAEs after ICI resumption. Among patients who experienced a recurrent irAE, 70% had their initial irAE, 25.7% had a *de novo* irAE, and 4.3% had both. Most patients (68.6%) required corticosteroids, and 8.6% required immunosuppressive medications. No deaths related to recurrent irAE were reported, and 76.6% of second irAEs resolved to grade 1 or lower (139).

There is a lack of data on the risk factors associated with irAE recurrence. Allouchery et al. found that initial gastrointestinal irAEs were more likely to recur (p = 0.007), whereas initial endocrine irAEs were associated with lower rates of irAE recurrence (p = 0.003) (139). Dolladille et al. reported that colitis (p = 0.01), hepatitis (p = 0.01), and pneumonitis (p = 0.01) were associated with a higher recurrence rate, whereas adrenal events were associated with a lower recurrence rate (p = 0.03) (140). However, Pollack et al. reported that colitis/diarrhea recurred less frequently than other irAEs (6% vs. 28%, p = 0.01) (141).

Abu-Sbeih et al. reported a multicenter retrospective study focusing on patients treated with ICI treatment after the onset of immune-mediated diarrhea and colitis (IMDC) (142). IMDC recurred in 34% of patients; 81% of the recurrences required corticosteroid therapy and 12% required the addition of infliximab or vedolizumab. IMDC recurred in 44% of patients who resumed CTLA-4 inhibitors and in 32% of whom resumed PD-1/L1 inhibitors. Multivariate analysis showed that risk factors significantly associated with IMDC recurrence were the initial need for immunosuppressive therapy (p = 0.019) and the longer duration of initial IMDC symptoms (p = 0.031) (142). Initial use of PD-1/L1 inhibitors was higher risk of IMDC recurrence than CTLA-4 inhibitors (p = 0.002). However, PD-1/L1 inhibitor resumption had lower risk of recurrence than CTLA-4 inhibitors resumption, regardless of the type of initial ICI (p = 0.019) (142). Pollack et al. studied 80 patients who discontinued PD-1 and CTLA-4 inhibitor combination therapy due to irAEs. Of these, 96% received corticosteroids and 21% received additional immunosuppressive treatment. All patients were resumed on PD-1 inhibitors, and patients with initial colitis or hypophysitis could safely resume PD-1 inhibitors (141). However, Allouchery et al. reported that the resumption of the same ICI treatment was associated with a lower rate of irAE recurrence (77.1% vs. 90%, p = 0.02) (139).

The reported duration before resumption ranged from 0.9 to 6.3 months (139–145). Sminonaggio et al. reported that a shorter time to the first irAE was associated with the occurrence of a second irAE (9 vs. 15 weeks, p = 0.04) (143). However, Allouchery et al. reported that the duration from ICI discontinuation to resumption and irAE severity did not predict recurrent irAEs (p = 0.53 and p = 0.40, respectively) (139).

To investigate the efficacy of resuming ICI retreatment, Santini et al. retrospectively analyzed 68 NSCLC patients with severe irAEs requiring interruption of PD-L1 inhibitor (144). Among them, 56% of patients resumed PD-L1 inhibitors, and 44% were permanently discontinued. In the retreatment cohort, 48% of patients had no irAEs, 26% had initial irAEs recurrence, and 26% had *de novo* irAEs. Most recurrences/*de novo* irAEs were mild (58% were grade 1-2) and controllable (84% resolved or reduced to grade 1). For those without an observed partial response before the irAE, PFS and OS were longer with ICI resumption. Conversely, in those who had an objective response before irAE, PFS and OS were similar with or without resumption of ICI. Therefore, patients who had no treatment response prior to irAE onset may be more need in ICI resumption. Similarly, another retrospective analysis of melanoma patients treated with nivolumab and ipilimumab showed that patients could continue to benefit from previous immunotherapy even after the discontinuation due to irAEs (145).

Overall, the retrospective design, heterogeneity of cancer type, irAE and ICI treatment, and small sample sizes of the studies make it difficult to conclude resumption after irAEs. AE recurrences are mostly low-grade and manageable, but it remains controversial whether there is any additional benefit from continuing ICI after irAE. The risk-benefit ratio of resumption should be considered personally. It is challenging but necessary to look for specific predictive risk factors for irAE recurrence.

### 6.3 Prevention of flares/irAEs with selective immunosuppressive medications

There are no successful strategies for preventing irAEs. Previous studies mentioned that selective antibody therapy, such as anti-IL-6, anti-CD20, and anti-TNF therapy, is effective in treating irAEs or preventing the flares and maintaining the clinical benefit of ICIs (146–148). This increased our interest in therapies that could control PAD and alleviate cancer at the same time.

Many medications for ADs also have anti-tumor activity. Janus kinase/signal transducer and activator of transcription (JAK/STAT) signaling regulates the secretion of various cytokines, which play a central role in the development of ADs and tumorigenesis (149). Inhibition of JAK/STAT pathways by JAK inhibitors alleviates ADs by decreasing cytokines secretion. Moreover, JAK inhibitors have the potential to counteract tumorigenesis by reversing drug resistance, inducing G2 arrest, and augmenting apoptosis (150).

In a phase II clinical trial, 21 treatment-naïve metastatic NSCLC patients with tumor PD-L1 ≥50% were treated with pembrolizumab and delayed itacitinib (a JAK1 inhibitor) (151). Patients received pembrolizumab until disease progression, and two cycles of itacitinib were added on cycles 3 and 4. The 12-week ORR was 62%, and the best overall response (BOR) after 12 weeks was 67%. After a median follow-up time of 27.6 months, the median PFS was 23.8 months. Response assessment at 6 weeks showed that five patients had an early response to pembrolizumab before itacitinib. In contrast, eight patients who failed to respond or progress after initial pembrolizumab were able to respond after itacitinib. Hence, patients who failed to respond to initial PD-1 inhibitor could still achieve a high response rate with the addition of a JAK inhibitor in NSCLC patients with tumor PD-L1 ≥50%. Itacitinib promoted CD8 T cell plasticity and therapeutic responses of exhausted and effector memory-like T cell clonotypes. Thus, it may enhance the efficacy of PD-1 inhibitors by altering the dynamics of T-cell differentiation.

### 6.4 Reduce toxicities via development of next-generation ICIs and drug-delivery system

Recent advances in protein engineering and drug delivery technologies are addressing the critical need to improve the safety profile of ICIs, particularly for cancer patients with PADs.

To enhance selectivity and minimize toxicity, protein engineered next-generation ICIs are being developed with optimized pharmacokinetics, including longer half-lives, greater stability, and more controlled activation (152). One strategy is to modify the Fc region of ICIs to reduce binding to Fcγ receptors and minimize off-target immune activation of macrophages or dendritic cells (153). For example, IgG4 isotype ICIs have lower Fc effector function than IgG1 type (154). Second, tumor-specific bispecific antibodies are designed with increased affinity to target tumor cells while sparing normal tissues. It could target ICIs specifically to tumors by conjugating to tumor antigen-binding domains (e.g., Anti-PD-1/Her2 Bispecific Antibody IBI315) (155). Third, modified immune checkpoint proteins are used to make ICIs inactive until conditionally active in the tumor microenvironment (TME). For example, CX-072 (a PD-L1 inhibitor) has protease-cleavable masking and is only activated by tumor-associated proteases (156). pH low insertion peptide (pHLIP) modified PD-L1 has pH-sensitive design and suppresses T-cell activation in the acidic TME (157). Hence, pHLIP modified ICIs could remaining less active in normal tissues and potent in the acidic TME. Selective targeting reduces off-target effects and minimizes the risk of irAEs, paving the way for safer cancer immunotherapies for cancer patients with PAD.

Otherwise, specific drug delivery systems are used for safer ICI therapy. Encapsulation of ICIs in nanoparticles, which release medications preferentially in the TME with the design of pH-, redox-, or enzyme-responsive, makes the medications tumor-targeted and reduces broad immune activation (158, 159). Cell membrane-coated nanoparticles use autologous immune cell membranes (e.g., T cell membranes) to improve tumor homing and reduce off-target effects (160).

Intriguingly, irAEs can be prevented to some extent by modifying ICIs so that they are only active within the TME, or restricting their delivery to the TME.

## 7 Conclusion

The increasing prevalence of PADs among lung cancer patients presents both challenges and opportunities for the use of ICIs. Our comprehensive review addresses critical gaps in the existing literature by incorporating the most recent clinical evidence (2021-2024) that was absent from previous reviews limited to pre-2021 data and predominantly melanoma-focused studies. The unique value of our work lies in several key contributions. First, we provide a mechanistic understanding of the relationship between autoimmune diseases and lung cancer, particularly examining how dysregulated immunity, chronic inflammation, and tissue damage in autoimmune conditions may contribute to oncogenesis. Second, we offer a comprehensive synthesis of clinical evidence regarding ICI safety and efficacy specifically in lung cancer patients with autoimmune comorbidities, including analysis of combination therapies with chemotherapy or dual ICIs. Third, our review advances translational research by exploring the relationship between immune-related adverse events and clinical outcomes, while discussing potential predictive biomarkers for risk stratification. We also develop a practical clinical decision framework to guide optimal timing and use of ICIs in this complex patient population. When considering ICIs, it’s important to weigh the potential benefits against the risks of exacerbating PAD or *de novo* irAEs. This balance may differ depending on the PAD activity, use of immunosuppressive treatments, type of PAD, ICI and cancer.

In conclusion, although ICIs present an exciting therapeutic option for lung cancer patients with PADs, their use requires individualized treatment strategies with multidisciplinary management. It is acceptable to use ICIs in patients with a low risk of irAE after informing patients of involved irAEs and monitoring them closely. Early recognition and timely intervention for irAEs can help ensure safer ICI administration. The resumption of ICI after irAEs is another clinical question that remains to be answered. Prevention of flare/irAE with selective immunosuppressive medications is an important area of future research.
